# Effectiveness and Tolerability of Anti-Calcitonin Gene-Related Peptide Therapy for Migraine and Other Chronic Headaches in Adolescents and Young Adults: A Retrospective Study in the USA

**DOI:** 10.3390/brainsci14090879

**Published:** 2024-08-30

**Authors:** Anjaneya Shankar Madhav Bandatmakur, Pooja Dave, Melissa Kerr, Colin Brunick, Sijin Wen, Nicholas Hansen

**Affiliations:** 1 Pediatric Neurologist-Headache Specialist, Department of Pediatric Neurosciences, Boys Town National Research Hospital, Omaha, NE 68010, USA; 2 Pediatric Hospitalist, Department of Pediatrics, Children’s Hospital & Medical Center, Omaha, NE 68114, USA; 3 Department of Epidemiology and Biostatistics, West Virginia University, Morgantown, WV 26506, USA; siwen@hsc.wvu.edu; 4 School for Environment and Sustainability, University of Michigan, Ann Arbor, MI 48109, USA

**Keywords:** pediatric headache, migraine, anti-CGRP, headache, anti-migraine treatment

## Abstract

This retrospective study assesses the efficacy and tolerability of anti-calcitonin gene-related peptide (anti-CGRP) therapy in adolescents and young adults (ages 12–21) with migraine and chronic daily headaches unresponsive to standard treatments. Migraines in this demographic significantly impair school performance, self-esteem, psychological well-being, and cognitive health. These young patients are also particularly sensitive to the side effects of conventional medications, which are often prescribed off-label and come with high insurance denial rates. Medication overuse, including analgesics, triptans, and NSAIDs, is prevalent due to treatment failures. Elevated plasma CGRP levels observed during migraines suggest that anti-CGRP therapies, successful in adult populations, may also benefit this younger age group. Over a three-year period, patients at a specialized pediatric headache center were evaluated for the impact of anti-CGRP treatments, including monoclonal antibodies (erenumab, fremanezumab, and galcanezumab) and small-molecule CGRP receptor antagonists (ubrogepant, rimegepant, and atogepant), administered either alone or in combination with OnabotulinumtoxinA. Data were extracted from the hospital’s electronic medical records, and patient progress was consistently documented using a structured template for each clinic visit. Additional patient satisfaction data were collected via telephone follow-ups and patient message reviews. The study included 23 patients, primarily treated for chronic migraine (CM) (78.3%), with a smaller subset addressing episodic migraine (EM), new daily persistent headaches (NDPHs), and post-traumatic headaches (PTHs). Comprehensive demographic and clinical data, including age, treatment duration, history of preventive treatment failures, and comorbidities like psychiatric conditions and sleep disorders, were collected. Anti-CGRP therapies, particularly when combined with traditional treatments or OnabotulinumtoxinA, resulted in significant improvements: 91.3% of patients experienced reduced migraine duration and intensity, 82.6% reported improvements in other bothersome symptoms, and 73.9% saw an improved response to rescue medications. Additionally, 78.3% of patients reported a reduction in their use of rescue medications per week by more than 50%, and emergency room visits were reduced for 56.5% of patients. Significant reductions in headache days were observed in 82.6% of patients after one month and 87% after three months, with nearly 40% experiencing more than a 50% reduction in both periods. The greatest benefits were observed in patients treated for more than six months. Adverse effects were minimal, with 95.7% of patients reporting no side effects, and patient satisfaction was high, with 69.6% opting to continue treatment. Overall, this study highlights the substantial potential of anti-CGRP therapy in improving outcomes for adolescents and young adults with CM and EM, offering a promising approach for a demographic that faces considerable challenges with conventional treatment options. However, further research is needed to confirm these findings and expand clinical applications in this age group.

## 1. Main Manuscript

**Introduction/Objective**: Migraine is a debilitating neurological condition significantly impacting adolescents and adults alike. This retrospective study evaluates the efficacy and tolerability of anti-calcitonin gene-related peptide (CGRP) therapy—encompassing monoclonal antibodies and small-molecule antagonists—in treating resistant migraine in individuals aged 12 to 21. These therapies are used either independently or in conjunction with OnabotulinumtoxinA to target migraine and other chronic headache forms that have not responded to conventional treatments.

**Background**: Migraine is prevalent among children and adolescents, significantly impacting their long-term school performance, self-esteem, psychological well-being, and cognitive health [1,2]. This presents a therapeutic challenge due to the limited availability of evidence-based prevention options [3]. The success of anti-calcitonin gene-related peptide (CGRP) monoclonal antibodies (mAbs) in preventing migraine and episodic cluster headaches in adults, coupled with minimal adverse events, has generated interest [4,5,6,7,8,9,10,11,12,13]. During migraine episodes, increased plasma levels of CGRP are noted in both adult [14] and pediatric patients [15,16], demonstrating a correlation with headache intensity [16,17]. In the pediatric context, elevated plasma CGRP levels not only differentiate migraine from non-migraine headaches but also serve as a biomarker for guiding drug therapy strategies [18]. Despite CGRP expression in various body systems [13], recent adult trials have shown minimal adverse events, primarily involving injection site reactions, upper respiratory symptoms, and constipation. These treatments offer an advantage by eliminating the need for daily pill consumption, potentially improving adherence, a common issue with oral migraine preventives [19]. However, extending this success to the younger demographic is hindered by the absence of definitive pediatric and adolescent outcome data, necessitating a careful examination of potential risks and benefits.

The Pediatric and Adolescent Headache special interest group advocates for a nuanced approach, suggesting the consideration of anti-CGRP mAbs in suitable cases. In 2018, Szperka et al. issued recommendations for the use of anti-CGRP monoclonal antibodies in children and adolescents with migraine [20]. In 2020, Greene et al. conducted the initial study reporting on the safety and efficacy of anti-CGRP mAb use in adolescents with chronic headache disorders. The retrospective multi-site cohort study included adolescents under 18 years with chronic migraine, NDPH, or persistent post-traumatic headache, two-thirds of whom experienced continuous headache at baseline. The findings suggest that anti-CGRP mAb treatment benefits a proportion of adolescents with chronic refractory headache disorders, and the observed side effects align with those reported in adult trials [21].

Expanding the scope to include prevalent headache types in children and adolescents, like new daily persistent headache (NDPH) [22,23] and post-traumatic headache (PTH) [22,24], reveals a therapeutic landscape lacking concrete evidence, necessitating cautious exploration [24,25]. For cases of persistent PTH and sometimes NDPH with a migrainous phenotype resistant to conventional therapies, the consideration of anti-CGRP mAbs is deemed reasonable, recognizing potential pathophysiological overlaps [20,21,24,26,27,28]. One study found that erenumab reduced headache frequency and improved psychological symptoms in chronic migraine patients, with a slight, non-significant shift in chronotype and reduced sleep efficiency observed [29].

In addition to monoclonal antibodies targeting the CGRP pathway (e.g., erenumab, eptinezumab, fremanezumab, and galcanezumab), another category of anti-CGRP medications includes small-molecule CGRP receptor antagonists known as gepants (e.g., rimegepant, ubrogepant, and atogepant), which have been introduced to the market [30]. Numerous clinical trials and real-world observational studies consistently affirm their efficacy and tolerability for both the acute and preventive treatment of migraine in adults [31]. While randomized controlled trial data for the pediatric population are pending for both types of medications, the American Headache Society (AHS) has provided recommendations for their use in pediatric headache disorders, as discussed previously [20].

Furthermore, OnabotulinumtoxinA was effective in reducing monthly headache days, and the addition of an anti-CGRP monoclonal antibody was safe, well-tolerated, and resulted in meaningful reductions in MHD for those continuing the combined treatment, with no new safety concerns identified [32,33]. While acknowledging therapeutic potential, a prevailing emphasis on caution is maintained due to the absence of long-term safety data for this age group, highlighting the need for further research and judicious clinical decision-making. The importance of robust trials cannot be overstated, both for informed therapeutic decisions and to advocate for improved access and insurance coverage for this age group.

This retrospective study assesses the efficacy and tolerability of anti-CGRP therapy in adolescents and young adults (ages 12–21) with migraine and chronic daily headaches unresponsive to standard treatments.

## 2. Methodology

This study is a retrospective chart review conducted at a dedicated pediatric headache clinic within the Boys Town National Research Hospital, Nebraska, USA. The study period spanned from October 2020 to December 2023. Approval for the study was obtained from the institutional review board of Boys Town National Research Hospital (protocol number: 23-22-X; date: 28 August 2023), and the requirement for written informed consent was waived due to the retrospective nature of the research. There was no external funding source for this study. Data were extracted from the hospital’s electronic medical record (EMR) system, specifically from EPIC EMR. A structured template (Figures S1 and S2) was employed for each clinic visit to document key elements pertinent to headache management, such as the duration and intensity of migraines. This template facilitated consistent monitoring of patient progress across visits. Patient satisfaction data were primarily collected using the clinic template and supplemented by telephone calls. Additionally, patient messages and phone calls were reviewed to gather comprehensive information. The results were systematically recorded for analysis. Patients were identified using a spreadsheet that tracked those receiving anti-CGRP therapy and those who underwent OnabotulinumtoxinA injections within the study period. These patients were either treated under the indicated guidelines for anti-CGRP therapy if they were over 18 or under off-label conditions if younger. These included anti-CGRP monoclonal antibodies targeting the CGRP pathway (erenumab, eptinezumab, fremanezumab, and galcanezumab) and small-molecule CGRP receptor antagonists (rimegepant, ubrogepant, and atogepant). Some patients also received Onabotulinumtoxin A in combination with anti-CGRP therapy.

The primary conditions targeted include episodic migraine (EM), chronic migraine (CM), new daily persistent headache (NDPH), chronic post-traumatic headache (PTH), and chronic daily headache (CDH). NDPHs and chronic PTHs showed migrainous characteristics despite their primary diagnoses. Several factors influenced the decision to initiate anti-CGRP therapy, including the duration of therapy usage, a history of unsuccessful first-line preventive migraine medications, prior medical history, such as concussions, psychiatric conditions, like anxiety, depression, PTSD, and ADHD, the presence of sleep disorders, and other coexisting neurological conditions such as epilepsy and developmental delays. Patients exclusively receiving abortive anti-CGRP therapies, such as rimegepant or ubrogepant for acute migraine attacks, were excluded from the study.

For each patient, demographic and clinical data were collected, including age group, indication for treatment, duration of use, first-line preventive failures, history of concussion, psychiatric conditions, sleep disorders, and other neurological problems. Additionally, data on the type of anti-CGRP therapy were collected, with some patients using monoclonal antibodies (mAbs), others using antagonists, and some using both. Information was also gathered on whether anti-CGRP therapy was combined with traditional therapy and the purpose of anti-CGRP therapy, whether for rescue, prevention, or both. Furthermore, the study tracked the use of anti-CGRP therapy combined with OnabotulinumtoxinA, response to rescue medications, reduction in rescue medication use, reduction in emergency room (ER) visits, reduction in the duration and intensity of migraines, improvement in other bothersome symptoms, reduction in the number of headache days after one month and three months, adverse effects, reasons for discontinuation, and patient satisfaction. The collected data were subjected to statistical analysis to evaluate the effectiveness and safety of anti-CGRP therapies and OnabotulinumtoxinA injections. Key outcomes included the reduction in the number and intensity of headache days after 1 and 3 months of therapy, the decrease in the use of rescue medications, and the reduction in ER visits. Adverse effects and reasons for discontinuation were also analyzed to assess the tolerability of the treatments. Patient satisfaction was also evaluated based on survey responses concerning the overall treatment experience.

This methodology outlines a comprehensive approach to evaluating the effectiveness of anti-CGRP therapies and OnabotulinumtoxinA injections in pediatric patients with CM. The structured data collection and thorough analysis aim to provide valuable insights into treatment outcomes and patient satisfaction, contributing to the optimization of headache management in this population.

Statistical analyses for this study were conducted using R software, version R 3.6.3, available at R Project. The primary objective was to assess the clinical outcomes associated with the use of anti-CGRP therapy (monoclonal antibodies and/or small-molecule antagonists) as a treatment for headaches. To achieve this, descriptive statistics summarized patient data, with categorical variables presented in contingency tables featuring counts and percentages and continuous variables described through means (±standard deviation) or medians (range). For inferential statistics, Fisher’s exact test was applied to categorical data and the Wilcoxon rank-sum test to continuous data to determine differences between groups. A *p*-value of less than 0.05 was considered statistically significant, guiding the interpretation of the study’s findings. In the power analysis using the statistical software PASS, version 23.0.3 (NCSS, LLC. Kaysville, UT, USA, Year 2023), a total of 23 patients in this study achieved 80% power to detect an effect size of at least 0.62 in the outcome variables of interest, using a two-sided paired t-test at a significance level of 0.05. Moreover, this sample size had 80% power to differentiate between the improvement rate of 0.50 (null) and 0.75 (alternative) based on the binomial distributions at a significance level of 0.05.

## 3. Results

This study offers a comprehensive analysis of the effectiveness and application of anti-CGRP monoclonal antibodies (mAbs) and antagonists in treating migraine across various age groups and conditions. A total of 23 patients were included in the study, predominantly young; 39.1% were between 12 and 15 years, 52.2% were aged between 16 and 18 years, and a smaller segment, 8.7%, were over 18 years old. Most of these patients were treated for CM, which accounted for 78.3% of the cases, while EM comprised 13% of the conditions treated. Less common conditions included new daily persistent headaches (NDPHs) and combinations of post-traumatic headaches. The two patients in these groups (NDPH and PTH) were included in the analysis because their headache characteristics closely aligned with migrainous phenotypes, despite their primary diagnoses.

The study also highlighted the complex comorbidities within the patient population, with 91.3% of the participants suffering from psychiatric conditions and 69.6% experiencing sleep disorders. Regarding their medication history, more than half of the patients (56.5%) had not found relief with one to two preventive medications, and 30.4% had not seen improvements with three to four medications (Table 1).

The treatment modalities involving anti-CGRP therapy among the patients were distributed as follows: 39.1% received anti-CGRP combined with traditional treatments, 26.1% were treated with anti-CGRP in conjunction with both OnabotulinumtoxinA and traditional treatments, 21.8% received only anti-CGRP therapy, and 13.0% were treated with anti-CGRP and OnabotulinumtoxinA. Anti-CGRP mAbs were the primary therapy used by 65.2% of the patients, while 13% received antagonists, and 21.7% were treated with a combination of both. Most of the patients (60.9%) used anti-CGRP treatments primarily for prevention, and 39.1% used them for both acute and preventive purposes. These treatments were typically utilized long-term, with 78.3% of patients having used them for over six months (Table 2). It was also noted that the patient group with more than 6 months of use had the most response to rescue medications (*p* = 0.003) and a significant reduction in ER visits (*p* = 0.007) due to a reduction in the duration and intensity of migraines (*p* = 0.043 and *p* = 0.057, respectively).

A total of 86.6% of the patients with anti-CGRP along with traditional therapy (*n* = 13) reported significant reduction in the duration of migraines (*p* = 0.058) and the intensity of migraines (*p* = 0.026). This group also had a notable reduction in the number of headache days after 3 months (*p* < 0.001).

The effectiveness of the treatments was notable; 91.3% of patients experienced reduced duration and intensity of migraines, and 82.6% reported improvements in other bothersome symptoms. Additionally, the response to rescue medications improved for 73.9% of patients, and 78.3% reported a reduction in their use of rescue medications per week by more than 50% due to an improvement in response (*p* = 0.08). There was a significant reduction in the number of headache days; 82.6% saw reductions after one month and 87% after three months, with nearly 40% of patients experiencing more than a 50% reduction in both periods. Emergency room visits were reduced for 56.5% of patients (*p* = 0.05).

Moreover, adverse reactions were minimal, with 95.7% of patients reporting no side effects.

The high level of patient satisfaction was reflected in the 69.6% of participants expressing strong satisfaction with their treatment outcomes (*p* = 0.038), including improved response to rescue medications (*p* = 0.045), reduction in ER visits (*p* = 0.019), reduction in duration (*p* = 0.021) and intensity (*p* = 0.056), improvement in other bothersome symptoms (*p* = 0.006), and significant decrease in the number of headache days (more than 50%) after 1 month (*p* = 0.021) and 3 months (*p* = 0.004). Similarly, the continuation rate was also high, with 69.6% opting to continue the treatment. These results underscore the significant potential of anti-CGRP therapies in effectively managing migraines, particularly among younger populations with complex comorbidity profiles (Table 3).

## 4. Discussion

Migraine remains a debilitating neurological condition that significantly impacts adolescents, often disrupting daily activities and diminishing their quality of life. The prevalence of migraines in adolescents is increasing, leading to significant academic, psychological, and social challenges [1,2]. This age group is often sensitive to the adverse effects and interactions of conventional migraine medications, many of which are prescribed off-label. In the USA, even among traditional anti-migraine treatments, only Topiramate has been approved for labeled use for migraine prevention in patients from 12 to 18 years. Among rescue medications, only four triptans—Almotriptan, Rizatriptan, Zolmitriptan ODT, and the Sumatriptan/Naproxen combination—are approved for labeled use. Additionally, high insurance denial or cost concerns due to non-coverage are prevalent. Medication overuse is a major issue with analgesics, triptans, and NSAIDs in this patient population due to treatment failures and resistance.

The advent of anti-CGRP therapies has revolutionized adult migraine management, yet their efficacy and safety in the pediatric demographic are not well documented. The limited pediatric-specific trials for these medications highlight the urgent need for evidence-based therapies tailored to this demographic [20].

This retrospective chart review study addresses these gaps by evaluating the application of anti-CGRP therapies in adolescents with chronic migraine (CM), chronic daily headache (CDH), new daily persistent headache (NDPH), and chronic post-traumatic headache (PTH). Patients in our study with NDPH and chronic PTH had headache characteristics that aligned with migrainous phenotypes, despite their primary diagnoses. Over three years, we analyzed treatment-resistant patients aged 12 to 21 at a specialized headache center, assessing the impact of anti-CGRP treatments, both as monotherapies and in combination with OnabotulinumtoxinA. Our findings indicate substantial improvements in migraine management, including reduced headache days, reduced use of rescue medications, fewer emergency room visits, improved functional outcomes in adolescents, and overall increased patient satisfaction. A longer course of therapy appears to be more effective, as indicated by the reduction in migraine burden and improved response to rescue medications. Additionally, we observed a better response with the combination of anti-CGRP and traditional therapy compared to either monotherapy alone. This improvement led to enhanced school functionality, increased participation in academic activities and sports, better sleep patterns, improvements in psychological conditions (e.g., less stress, better mood, and improved concentration and focus), and fewer school absences.

These findings highlight the effectiveness of anti-CGRP therapy in managing migraines among adolescents, a demographic notoriously difficult to treat due to the variability in migraine pathology and treatment response. The therapy showed significant efficacy across various migraine types without correlation with psychiatric comorbidities or prior medication failures. These observations suggest that anti-CGRP therapy could be broadly applicable and effective, warranting its consideration in personalized treatment plans for adolescents.

Our findings also align with recent studies suggesting the efficacy of anti-CGRP therapies in adolescents. The American Headache Society and various special interest groups have tentatively recommended these therapies, backed by emerging data from cohort studies [20]. For instance, Greene et al. (2020) noted comparable safety and efficacy in a similar demographic [21]. Our study extends these findings by confirming the substantial therapeutic benefits of combining anti-CGRP therapies with traditional migraine medications, emphasizing a synergistic effect that enhances clinical outcomes.

Furthermore, we were able to take this study one step further by also assessing the efficacy of the combination of anti-CGRP therapy along with OnabotulinumtoxinA; so far, the only retrospective review to achieve so in this age group. We found that OnabotulinumtoxinA was effective in reducing monthly headache days, and the addition of an anti-CGRP monoclonal antibody was well tolerated and resulted in meaningful reductions in monthly headache days for those continuing the combined treatment, with no new safety concerns identified.

Adolescents are underrepresented in migraine research, yet they stand to benefit significantly from advances in treatment strategies. Effective management of migraine in this age group can positively impact academic performance, social interactions, and overall development and reduce the migraine burden into adulthood. Our study provides compelling evidence that anti-CGRP therapy can transform migraine management in adolescents, offering a new horizon in personalized medicine for this age group. The data suggest that these therapies, especially when used for extended periods and in combination with other treatments, could significantly improve clinical outcomes, highlighting the necessity for their consideration in routine clinical practice. Additionally, they were well tolerated with minimal adverse effects, aligning with findings from adult studies.

Despite the promising outcomes, our study stresses the importance of cautious interpretation and the need for further research to establish long-term safety and efficacy. The absence of long-term data in adolescents calls for careful clinical judgment and monitoring. The promising results of this retrospective study advocate for well-designed prospective clinical trials to validate and expand upon our findings. Such research is essential to solidify the role of anti-CGRP therapy in pediatric migraine management and to support its inclusion in treatment guidelines. Expanding research efforts to include this demographic is crucial to developing tailored and effective therapeutic options.

## 5. Conclusions

In conclusion, this retrospective study underscores the promising role of anti-CGRP therapy in managing migraine disorders among adolescents and young adults, a group traditionally underrepresented in migraine research. Notably, the therapy has shown beneficial effects in those unresponsive to conventional treatments, with marked improvements in patient satisfaction, reduction in migraine frequency, and enhanced overall quality of life. Our findings suggest that the combination of anti-CGRP therapy with traditional treatments or OnabotulinumtoxinA could offer a synergistic benefit, enhancing the therapeutic outcomes and tolerability. This retrospective study contributes valuable insights into the potential of anti-CGRP therapies in managing migraine in this age group.

While the results are promising, they underscore the need for ongoing research and careful clinical application. Future trials are essential to confirm these findings and advocate for broader access and insurance coverage for effective migraine management in this vulnerable population. The therapeutic potential is substantial but must be approached with prudence due to the current gaps in long-term safety data.

## Figures and Tables

**Table 1:** Results Table 1.

Column (Variables)	Summary
**Age Group**	***n*—out of 23 (%)**
12–15 Y	9 (39.1)
16–18 Y	12 (52.2)
>18 Y	2 (8.7)
**Indication**	
Episodic Migraine (EM)	3 (13)
Chronic migraine (CM)	18 (78.3)
NDPH (CM)	1 (4.3)
Post-traumatic (CM, NDPH)	1 (4.3)
**1st line preventive failures**	
1–2 meds	13 (56.5)
3–4 meds	7 (30.4)
>4 meds	3 (13)
**History of concussion**	
Yes	8 (34.8)
No	15 (65.2)
**Psychiatric conditions**	
Yes	21 (91.3)
No	2 (8.7)
**Sleep disorder**	
Yes	16 (69.6)
No	7 (30.4)
**Other neurological problems**	
Yes	5 (21.7)
No	18 (78.3)

**Table 2:** Results Table 2.

Column (Variables)	Summary
**Treatment**	***n*—out of 23 (%)**
Anti-CGRP + Traditional	9 (39.1)
Anti-CGRP + OnabotulinumtoxinA	3 (13)
Anti-CGRP + OnabotulinumtoxinA + Traditional	6 (26.1)
Anti-CGRP alone	5 (21.8)
**Months of use**	
<3 months	3 (13)
4–6 months	2 (8.7)
>6 months	18 (78.3)
**Anti-CGRP therapy for rescue or prevention or both**	
Rescue	
Prevention	14 (60.9)
Both	9 (39.1)

**Table 3:** Results Table 3.

Column (Variables)	Summary
**Response to rescue meds**	***n*—out of 23 (%)**
Improved	17 (73.9)
No change	6 (26.1)
**Reduction in rescue med use in a week**	
Yes (>50%)	18 (78.3)
No (<50%)	5 (21.7)
**ER visit reduction**	
Yes	13 (56.5)
No	10 (43.5)
**Duration of migraine reduced**	
A lot	7 (30.4)
Some	14 (60.9)
None	2 (8.7)
**Intensity of migraine reduced**	
A lot	9 (39.1)
Some	12 (52.2)
None	2 (8.7)
**Other bothersome symptom improvement**	
A lot	9 (39.1)
Some	10 (43.5)
None	4 (17.4)
**Number of headache days reduction after 1 month**	
>50%	9 (39.1)
<50%	10 (43.5)
same	4 (17.4)
**Number of headache days reduction after 3 months**	
>50%	9 (39.1)
<50%	11 (47.8)
Same	3 (13)
**Adverse effects**	
Few	1 (4.3)
None	22 (95.7)
**Discontinued for any reason**	
Yes	7 (30.4)
No	16 (69.6)
**Patient satisfaction**	
Strongly	16 (69.6)
Doesn’t matter	5 (21.7)
No	2 (8.7)

## Data Availability

Data are contained within the article or Supplementary Materials.

## Supplementary Materials

The following supporting information can be downloaded at https://www.mdpi.com/article/10.3390/brainsci14090879/s1, Figure S1: Headache note template part 1; Figure S2: Headache note template part 2.
