# Supplementary material for: Effectiveness and Tolerability of Anti-Calcitonin Gene-Related Peptide Therapy for Migraine and Other Chronic Headaches in Adolescents and Young Adults: A Retrospective Study in the USA
Source: Brain Sci. 2024 Aug 30;14(9):879. doi: 10.3390/brainsci14090879 (PMC11429543; doi:10.3390/brainsci14090879)
Supplement: Supplementary file 1 [file brainsci-14-00879-s001.zip › brainsci-3166712-supplementary.pdf]

HEADACHE TEMPLATE

HPI: [Patient] is a [age] years old [gender] presenting for evaluation and assistance with management of headaches.

Headache HPI:

- Onset:
- Location/radiation:
- Description:
- Intensity:
- Typical migraine associated symptoms:
- Other associated symptoms:
- Aura:
- Rapidity of onset:
- Typical duration of untreated/inadequately treated headache:
- Does it go beyond 1 day frequently?
- Worst time of day:
- Awaken from sleep:
- Early morning headaches:
- Seasonal pattern:
- 'Clustering' of headaches over time:
- Autonomic cephalalgia symptoms:
- Other craniofacial pain syndrome features:
- Signs of increased ICP**:**

Headache Trend:

	Initial frequency	Current frequency
Total headache days/month		
Tension-type headaches/month		
Migraines/month		
Pattern		

Overall pattern since problem began: improved/same/worse
Other relevant medical problems:
Additional Relevant History:

- History of head/neck trauma:
- History of head/neck surgery:
- Family history of headache problems:
- Use of meds that might worsen headaches:
- Exposure to carbon monoxide:
- Substance use:

Typical Precipitants or Aggravating Factors:
Risk factors identified so far for developing headaches include:
Lifestyle Factors Affecting

Figure S1. Headache note template Part 1

MEDICATIONS TO TREAT HEADACHES

Abortive meds:

- **Using triptan:** Yes/No
- **Using Ergots:** Yes/No
- **Using Opioids:** Yes/No
- **Combination analgesics:** Yes/No

Abortive Medication Efficacy:

Medication name	Scale of efficacy: 0 - No relief to 5 - Resolves a headache (per patient)

Prophylactic Medications:

- **In the past:**
- **Currently:**
- **Any improvement:**

Previous Rescue Failures:
Previous Preventive Failures:
Future Rescue Options:
Future Preventive Options:

Satisfaction/Degree of Functional Impairment:

- **School attendance or other daily activities affected by headaches:**
- **School functionality**
  - **Academic performance:**
  - **Participation in Sports:**
- **Sleep improvement:**
- **Improvement in psychological conditions:**

Figure S2. Headache note template Part 2.
